# College students' underlying perceptions of COVID-19 threat, healthcare discrimination, and healthcare system inequities associated with self-rated health across racial/ethnic groups in the U.S.

**DOI:** 10.3389/fpubh.2022.1028344

**Published:** 2023-01-06

**Authors:** Jessica R. Fernandez, Juliana S. Sherchan, Yong Ju Cho, Judy Nanaw, Nataria T. Joseph, Allana T. Forde

**Affiliations:** ^1^Division of Intramural Research, National Institute on Minority Health and Health Disparities, National Institutes of Health, Bethesda, MD, United States; ^2^Department of Psychology, University of Maryland, College Park, MD, United States; ^3^Division of Social Science, Seaver College, Pepperdine University, Malibu, CA, United States

**Keywords:** college students, race/ethnicity, COVID-19 threat, healthcare discrimination, U.S. healthcare system inequities

## Abstract

**Background:**

COVID-19-related health perceptions may differentially impact college students' stress, and in turn, their mental and physical health. This study examined racial/ethnic differences in college students' underlying perceptions of COVID-19 threat, healthcare discrimination, and U.S. healthcare system inequities and their associations with self-rated mental and physical health.

**Methods:**

Four-hundred-thirty-two university students completed an online survey (December 2020–December 2021). Latent class analyses identified classes of perceived COVID-19 threat (i.e., severity, susceptibility), healthcare discrimination, and U.S. healthcare system inequities. Regression analyses examined whether class membership varied by race/ethnicity and was associated with self-rated mental and physical health.

**Results:**

Class 1 members (27.3% of the sample) were more likely to identify as Hispanic or Latino, Non-Hispanic Asian, Non-Hispanic Black or African American, and Non-Hispanic Multiracial vs. Non-Hispanic White (vs. Class 4). Class 1 had high perceived COVID-19 threat, medium perceived healthcare discrimination, and high perceived U.S. healthcare system inequities, as well as higher odds of poorer mental and physical health (vs. Class 4).

**Conclusions:**

College students' underlying perceptions of COVID-19 threat, healthcare discrimination, and U.S. healthcare system inequities were associated with poorer health. Given that students with these perceptions were more likely to belong to minoritized racial/ethnic groups, concerns over COVID-19 risk and healthcare may partially explain racial/ethnic disparities in college students' health. This study contributes to a limited body of evidence on college students' perceptions of the U.S. healthcare system and suggests important ways that structural inequalities and racial/ethnic disparities in COVID-19 risk, healthcare discrimination, and concerns over U.S. healthcare system inequity may affect college students' health.

## Introduction

College campuses in the United States (U.S.) have been one of the highest-risk settings for SARS-CoV-2 viral transmission during the Coronavirus disease 2019 (i.e., COVID-19) pandemic (1). In 2020, close to 400,000 cases of COVID-19 infection were reported at 1,800 colleges and universities in the U.S. (2). More recently, after gradual declines in national COVID-19 cases, COVID-19 cases rose nationally by 61% in April 2022 and colleges across the U.S. reinstated indoor mask mandates and/or limited large gatherings (3, 4).

Amid these national COVID-19 outbreaks, college students across the U.S. reported heightened levels of stress [e.g., high levels of worry and/or emotional distress related to COVID-19 (5)]. In addition, COVID-19-related stressors disproportionately affected students from minoritized racial/ethnic groups1 [e.g., increased discrimination among Asian American students (6), high risk of losing someone close to them due to COVID-19 for students from Black, Latinx, and Pacific Islander communities (7, 8), high proportions of grief/loss reported by students from American Indian/Alaska Native and Native Hawaiian or Pacific Islander communities (9)]. Consequently, recent studies emphasized the importance of examining racial/ethnic disparities in college students' stress during the COVID-19 pandemic (10, 11).

Previous studies identified the fear of COVID-19 infection (e.g., fear of contracting the virus, concerns over its spread, high perceived COVID-19 severity and/or susceptibility) as a major driver of college students' COVID-19-related stress (12–14). Moreover, prior studies revealed racial/ethnic differences in college students' COVID-19 fears (14, 15) and suggested that racial/ethnic structural inequalities in the U.S. (e.g., disparities in housing, working conditions, and/or healthcare access) likely exacerbated COVID-19 fears among students from minoritized racial/ethnic groups by contributing to racial/ethnic disparities in COVID-19 exposure, morbidity, and mortality (15, 16). Considering that COVID-19-related stressors can exist at multiple socio-ecological levels [e.g., intrapersonal risk perceptions, interpersonal stressors, structural/system-level concerns (17)], college students' COVID-19-related stress may be influenced by concerns that span these levels (collectively referred to as “health-related perceptions”). These perceptions include fear of COVID-19 infection, the potential stress of engaging with health care providers due to experiences of healthcare discrimination, and concerns over U.S. healthcare system inequities [e.g., racial/ethnic disparities in treatment, access to testing, and/or distribution of the COVID-19 vaccine (18–20)].

Moreover, prior research suggested that college students' health-related perceptions can serve as stressors that influence both their mental and physical health. Among Chinese college students, fears of contagion were associated with poorer psychological and physiological health through higher perceived stress (21). Among college students in the U.S., fears of COVID-19 were associated with increased panic, worry and/or anxiety/depression (22, 23). However, with respect to racial/ethnic differences in college students' health, the evidence is mixed [e.g., whereas some studies found poorer mental health among students who identified as Hispanic or Latinx, Non-Hispanic Black, or Non-Hispanic Asian compared to Non-Hispanic White (5, 24), other studies did not reveal racial/ethnic differences in college students' mental and/or physical health (14, 25)].

These discrepancies may be partially attributed to measurement (e.g., measuring mental health as diagnoses of anxiety and/or depression may only capture stress for students who used health services, measuring physical health using COVID-19 diagnoses may not capture other ways stress can influence physical health). Importantly, there is a lack of research examining college students' health-related perceptions and global measures of self-rated health, despite prior findings that self-rated health was associated with college students' systemic inflammation (26) and was a robust predictor of future morbidity and mortality in adult populations (27). It is plausible that self-rated health may capture perceived changes in health prior to formal diagnoses (26) and could reveal important racial/ethnic differences in college students' stress and health.

Furthermore, much of the evidence on health-related perceptions and college students' health focused on mental health with limited studies on physical health, particularly for college students in the U.S. In addition, most studies focused on fear of the COVID-19 virus as a stressor, and did not include U.S. college students' perceptions of healthcare discrimination and U.S. healthcare system inequities (28, 29). Although one recent study addressed this research gap by examining college students' perceived COVID-19 severity and susceptibility, perceived healthcare discrimination, and perceived U.S. healthcare system inequities (28), the study outcomes included COVID-19 preventive behaviors (i.e., intentions to wear a face mask, social distance, and receive the COVID-19 vaccine). No studies to date examined how these health-related perceptions affect college students' mental and physical health and whether underlying health-related perceptions vary across racial/ethnic groups.

The present study addressed these gaps in the literature by conducting latent class and logistic regression analyses in a population of college students in the U.S. to (i) identify latent classes of health-related perceptions, (ii) examine racial/ethnic differences in latent class membership, and (iii) assess whether these latent classes were associated with college students' self-rated mental and physical health.

## Methods

### Data source

This study used data from the *Weighing Factors in COVID Health Decisions* survey conducted from December 2020 to December 2021 at the University of Maryland, College Park. College students who were 18 years of age or older completed a self-administered online survey using Qualtrics software and received university course credit for their participation. Informed consent was obtained using an electronic consent form and study procedures were approved by the University of Maryland, College Park Institutional Review Board.

Survey responses were collected from 491 students. Surveys were excluded from the analysis if they were incomplete (*n* = 38) or due to small samples within sociodemographic categories (*n* = 21, see “Race/ethnicity and sociodemographic covariates” under *Measures)*. The final analysis included 432 students.

### Measures

#### Latent class indicators

Drawing on recent latent class analyses (28), latent classes were generated using six indicators related to perceived COVID-19 severity and susceptibility, healthcare discrimination, and U.S. healthcare system inequities (28) (Supplementary Table S1). Consistent with prior Health Belief Model studies (20, 21), perceived COVID-19 severity and susceptibility (i.e., collectively referred to as “perceived COVID-19 threat”) were measured. Perceived COVID-19 severity was measured using the item “You believe COVID-19 is serious and life threatening” and perceived susceptibility was measured using the item “You are concerned about contracting COVID-19”. Response options for both items included “Very true,” “Somewhat true,” and “Not true” and were dichotomized (i.e., “Not true” and “Somewhat true” coded as 0; “Very true” coded as 1).

Perceived healthcare discrimination was measured using a 7-item modified version of the Everyday Discrimination Scale for health care settings (i.e., how often students encountered situations when receiving health care due to their race or ethnicity such as “Treated with less respect than other people” or “Felt like a doctor or nurse was not listening to what you were saying”) (23, 24). Students' responses (i.e., “Never,” “Once,” “2–3 times,” “4 times or more”) were dichotomized (i.e., mean scores of 0: no experiences of healthcare discrimination coded as 0; mean scores > 0: one or more experiences of healthcare discrimination coded as 1).

Perceived U.S. healthcare system inequities included three items related to treatment, access to COVID-19 testing, and distribution of the COVID-19 vaccine (30). Perceived treatment of COVID-19 patients from minoritized racial/ethnic groups was measured using the item “How often have racial and ethnic minority patients with COVID-19 been treated unfairly by the U.S. healthcare system because of their race or ethnicity?” with response options of “Very often,” “Somewhat often,” and “Never” dichotomized (i.e., “Never” coded as 0; “Somewhat often” and “Very often” coded as 1). Perceived access to COVID-19 testing for minoritized racial/ethnic groups was measured using the item “How true is it that racial and ethnic minority groups have less access to COVID-19 testing compared to Whites?” with response options of “Very true,” “Somewhat true,” and “Not true” dichotomized (i.e., “Not true” coded as 0; “Somewhat true” and “Very true” coded as 1). Perceived distribution of the COVID-19 vaccine across racial/ethnic groups was measured using the item “How confident are you that the COVID-19 vaccine will be distributed fairly across racial and ethnic groups?” with response options of “Very confident,” “Somewhat confident,” and “Not confident” dichotomized (i.e., “Very confident” coded as 0; “Somewhat confident” and “Not confident” coded as 1).

#### Self-rated mental and physical health

Self-rated mental and physical health were measured using the items “In general, how would you rate your overall mental health?” and “In general, how would you rate your overall physical health?”, respectively. Response options for each item included “Poor,” “Fair,” “Good,” “Very Good,” and “Excellent.” (31, 32) and were dichotomized (i.e., “Poor” and “Fair” coded as 0; “Good,” “Very Good,” and “Excellent” coded as 1). A combined self-rated mental and physical health item was also created: (i) good to excellent mental health and good to excellent physical health; (ii) good to excellent mental health and poor to fair physical health or good to excellent physical health and poor to fair mental health; and (iii) poor to fair mental health and poor to fair physical health.

#### Race/ethnicity and sociodemographic covariates

Students self-identified their race and ethnicity in two separate items. Students selected their race from the U.S. Census categories allowing for multiple selections of “American Indian or Alaska Native,” “Asian,” “Native Hawaiian or Other Pacific Islander,” “Black or African American,” “White,” and “Other” (i.e., with the option to fill-in another race when selecting “Other”). Students self-identified Hispanic ethnicity as “Hispanic or Latino” or “Not Hispanic or Latino”. Students' responses to the race and ethnicity items were combined into the categories of “Non-Hispanic Asian,” “Non-Hispanic Black or African American,” “Non-Hispanic Multiracial,” “Non-Hispanic White,” and “Hispanic or Latino.” Students who self-identified as Non-Hispanic Other Race (*n* = 12) were not included in the analysis given the small sample size. Students self-identified their gender as male, female, transgender male, transgender female, gender non-conforming, or gender “not listed”. Students who identified as transgender (*n* = 1), gender non-conforming (*n* = 6), or indicated their gender was “not listed” (*n* = 3) were not included in the analysis given small sample sizes. Students' gender identity (i.e., male, female) and household income (i.e., <$50,000, $50,000–99,999, >$100,000) were included as categorical covariates and age in years was included as a continuous covariate.

### Analyses

Chi-square and Analysis of Variance tests were conducted using R version 4.1.2 to describe sociodemographic characteristics of the study population and group differences when stratified by race/ethnicity. Latent class analysis was conducted in M*plus* Version 8.6 (25) to identify latent classes of students' health-related perceptions, assess racial/ethnic differences in latent class membership, and examine whether latent classes were associated with self-rated mental and physical health.

#### Identification of latent class health-related perceptions

Robust maximum likelihood estimation was used to generate a series of latent class models from the six latent class indicators. Stepwise model comparisons using sample-size adjusted Bayesian Information Criterion (SA-BIC), entropy, Lo-Mendell Rubin (LMR) likelihood ratio tests, and bootstrapped likelihood ratio tests were used to select the best fitting solution. Direct effects of the sociodemographic covariates on the latent class indicators were assessed in the final step of model selection (i.e., comparing SA-BIC across models) to test for measurement invariance (26).

#### Racial/ethnic differences in latent class membership

Multinomial logistic regression was used to assess racial/ethnic differences in latent class membership. A three step approach estimated latent classes while accounting for individuals' fractional probabilities of membership in more than one class (33). Latent class membership was regressed on race/ethnicity, household income, age, gender, and survey completion date (i.e., the month in which students completed the study; included as a continuous variable). Odds ratios (ORs) with 95% confidence intervals (CIs) were used to assess the conditional probabilities of each covariate being present within the latent class.

#### Associations between latent class health-related perceptions and self-rated mental and physical health

Logistic regression models using ORs and 95% CIs were used to examine the associations between the latent classes and mental and physical health. First, self-rated mental health and physical health were each regressed separately on latent class membership using the three-step estimation approach (33), adjusting for direct effects of race/ethnicity, household income, gender, age, and survey completion date on mental and physical health. Next, ordinal logistic regression, adjusting for covariates, examined the association between the latent classes and the combined categorical outcome of mental and physical health.

## Results

Most students identified as Non-Hispanic White (49.5%), identified as female (75.0%), had a household income >$100,000 (53.9%), and were on average 19.3 years of age (Table 1). There were significant differences across race/ethnicity observed for gender (*p* < 0.01), and household income (*p* < 0.01) (Table 1).

**Table 1:** Characteristics of the study population.

	**Total population** ***N* = 432**	**Hispanic or Latino** ***n* = 50 (11.6)**	**Non-Hispanic Asian** ***n* = 89 (20.6)**	**Non-Hispanic Black or African American** ***n* = 49 (11.3)**	**Non-Hispanic Multiracial** ***n* = 30 (7.0)**	**Non-Hispanic** **White** ***n* = 214 (49.5)**	***p*-value**
**Age (in years)**							0.50
Mean	19.3 (±1.3)	19.0 (±1.2)	19.4 (±1.4)	19.4 (±1.4)	19.2 (±0.9)	19.4 (±1.4)	
Min.-Max.	18–27	18–23	18–24	18–23	18–21	18–27	
**Gender**							< 0.01
Male	108 (25.0)	11 (22.0)	35 (39.3)	16 (32.7)	7 (23.3)	39 (18.2)	
Female	324 (75.0)	39 (78.0)	54 (60.7)	33 (67.3)	23 (76.7)	175 (81.8)	
**Household income**							< 0.01
< 50,000	68 (15.8)	14 (28.0)	21 (23.6)	14 (28.6)	2 (6.7)	17 (8.0)	
$50,000–99,999	131 (30.3)	17 (34.0)	25 (28.1)	22 (44.9)	7 (23.3)	60 (28.0)	
> $100,000	233 (53.9)	19 (38.0)	43 (48.3)	13 (26.5)	21 (70.0)	137 (64.0)	
**Perceived COVID-19 severity**							0.31
Not true/somewhat true	91 (21.4)	6 (12.0)	19 (21.6)	9 (18.4)	5 (16.7)	52 (24.9)	
Very true	335 (78.6)	44 (88.0)	69 (78.4)	40 (81.6)	25 (83.3)	157 (75.1)	
**Perceived COVID-19 susceptibility**							< 0.01
Not true/somewhat true	259 (60.1)	22 (44.0)	45 (50.6)	25 (51.0)	14 (46.7)	153 (71.8)	
Very true	172 (39.9)	28 (56.0)	44 (49.4)	24 (49.0)	16 (53.3)	60 (28.2)	
**Perceived healthcare discrimination**							< 0.01
No experiences	311 (72.3)	30 (60.0)	56 (63.6)	17 (34.7)	23 (76.7)	185 (86.9)	
>1 experiences	119 (27.7)	20 (40.0)	32 (36.4)	32 (65.3)	7 (23.3)	28 (13.1)	
**Perceived unfair treatment of COVID-19**							0.57
**patients from minoritized racial/ethnic groups**							
Never	48 (11.2)	5 (10.0)	13 (14.8)	4 (8.2)	5 (16.7)	21 (9.9)	
Somewhat/very often	381 (88.8)	45 (90.0)	75 (85.2)	45 (91.8)	25 (83.3)	191 (90.1)	
**Perceived less access to COVID-19 testing**							0.21
**for minoritized racial/ethnic groups vs. white groups**							
Not true	77 (17.9)	6 (12.0)	22 (25.0)	11 (22.4)	5 (16.7)	33 (15.4)	
Somewhat/very true	354 (82.1)	44 (88.0)	66 (75.0)	38 (77.6)	25 (83.3)	181 (84.6)	
**Perceived low confidence in fair distribution**							0.37
**of COVID-19 vaccine across racial/ethnic groups**							
Very confident	58 (13.4)	6 (12.0)	10 (11.2)	3 (6.1)	4 (13.3)	35 (16.4)	
Somewhat/not confident	374 (86.6)	44 (88.0)	79 (88.8)	46 (93.9)	26 (86.7)	179 (83.6)	
**Self-rated mental health**							0.99
Poor to fair	249 (57.6)	28 (56.0)	50 (56.2)	29 (59.2)	18 (60.0)	124 (57.9)	
Good to excellent	183 (42.4)	22 (44.0)	39 (43.8)	20 (40.8)	12 (40.0)	90 (42.1)	
**Self-rated physical health**							0.68
Poor to fair	389 (90.0)	44 (88.0)	77 (86.5)	45 (91.8)	28 (93.3)	195 (91.1)	
Good to excellent	43 (10.0)	6 (12.0)	12 (13.5)	4 (8.2)	2 (6.7)	19 (8.9)	
**Self-rated mental and**							0.49
**physical health (combined)**							
Good to excellent mental & physical	243 (56.2)	27 (54.0)	50 (56.2)	26 (53.1)	18 (60.0)	122 (57.0)	
Good to excellent mental & poor to	152 (35.2)	18 (36.0)	27 (30.3)	22 (44.9)	10 (33.3)	75 (35.0)	
fair physical or good to excellent physical & poor to fair mental							
Poor to fair mental & physical	37 (8.6)	5 (10.0)	12 (13.5)	1 (2.0)	2 (6.7)	17 (7.9)	
							

Results presented include *n* (%) or mean (±standard deviation). *P*-values are based on χ2 and ANOVA tests of group differences across race/ethnicity.

### Identification of latent class health-related perceptions

**Figure 1:**
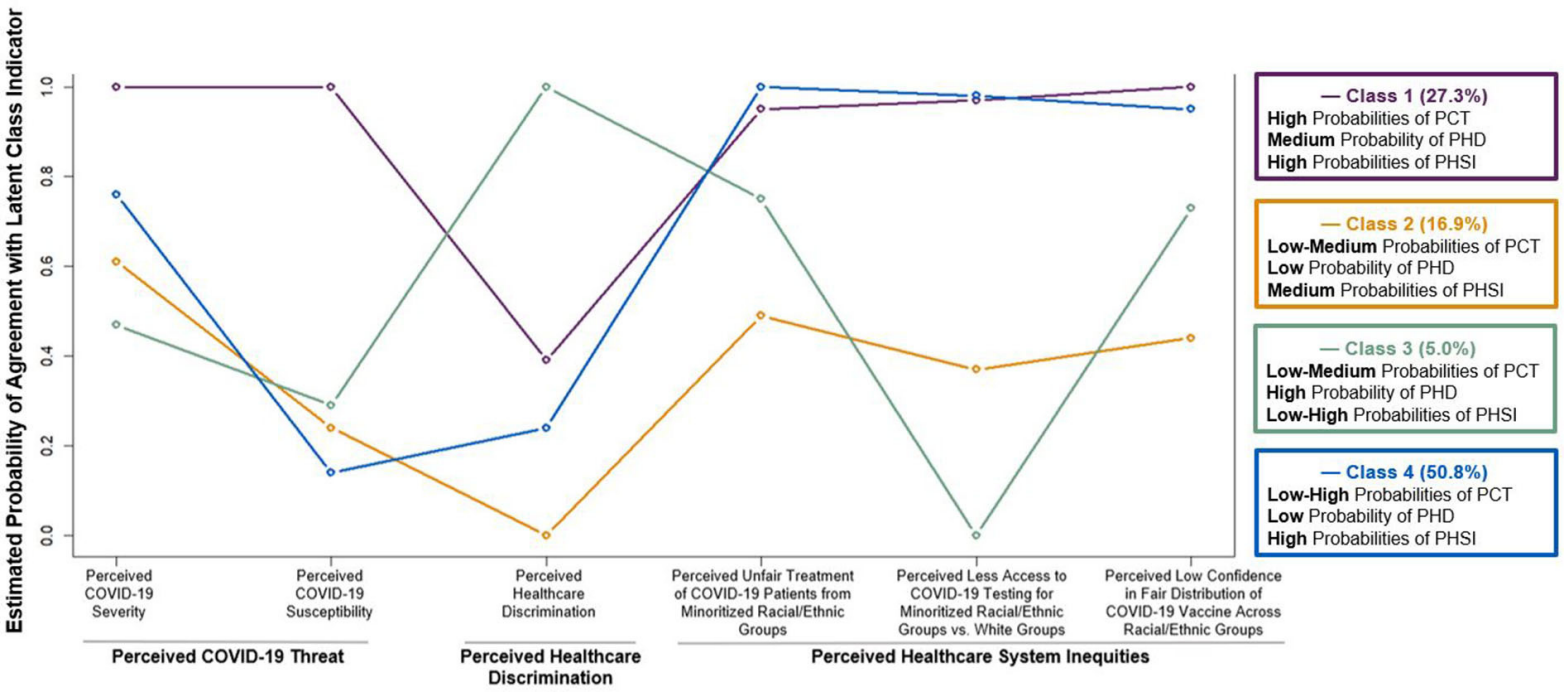
Four-class solution of perceived COVID-19 threat (PCT), perceived healthcare discrimination (PHD), and perceived healthcare system inequities (PHSI). Estimated probabilities of selecting 1 vs. 0 for each dichotomously coded latent class indicator. The classes were labeled using the estimated probabilities of perceived COVID-19 threat, perceived healthcare discrimination, and perceived healthcare system inequities which were categorized as low: <33%, medium: 33–66%, high: >66%.

The four-class solution was selected as the best fitting model (Supplementary Table S2). The model without direct effects of the sociodemographic covariates on the indicators had lower SA-BIC than the model including these direct effects, therefore the final model did not include the direct effects. Classes were labeled using the estimated probabilities of perceived COVID-19 threat, healthcare discrimination, and U.S. healthcare system inequities (Table 2; Figure 1). Students in Class 1 (27.3% of the sample) had *high* probabilities of perceived COVID-19 threat, a *medium* probability of perceived healthcare discrimination, and *high* probabilities of perceived U.S. healthcare system inequities. Students in Class 2 (16.9%) had *low to medium* probabilities of perceived COVID-19 threat, a *low* probability of perceived healthcare discrimination, and *medium* probabilities of perceived U.S. healthcare system inequities. Students in Class 3 (5.0%) had *low to medium* probabilities of perceived COVID-19 threat, a *high* probability of perceived healthcare discrimination, and *low to high* probabilities of perceived U.S. healthcare system inequities. Students in Class 4 (50.8%) had *low to high* probabilities of perceived COVID-19 threat, a *low* probability of perceived healthcare discrimination, and *high* probabilities of perceived U.S. healthcare system inequities.

**Table 2:** Estimated probabilities of agreement with latent class indicators.

	**Class 1**	**Class 2**	**Class 3**	**Class 4**
	**High probabilities of PCT**	**Low-medium probabilities of PCT**	**Low-medium probabilities of PCT**	**Low-high probabilities of PCT**
	**Medium probability of PHD** **High probabilities of PHSI** ***n* = 118 (27.3%)**	**Low probability of PHD** **Medium probabilities of PHSI** ***n* = 73 (16.9%)**	**High probability of PHD** **Low-high probabilities of PHSI** ***n* = 22 (5.0%)**	**Low probability of PHD** **High probabilities of PHSI** ***n* = 219 (50.8%)**
**Latent Class Indicators**				
**Perceived COVID-19 threat (PCT)**				
Perceived COVID-19 severity	1.00	0.61	0.47	0.76
Perceived COVID-19 susceptibility	1.00	0.24	0.29	0.14
**Perceived healthcare discrimination (PHD)**	0.39	0.00	1.00	0.24
**Perceived healthcare system inequities (PHSI)**				
Perceived unfair treatment of COVID-19 patients from minoritized racial/ethnic groups	0.95	0.49	0.75	1.00
Perceived less access to COVID-19 testing for racial and ethnic minoritized racial/ethnic groups vs. white groups	0.97	0.37	0.00	0.98
Perceived low confidence in fair distribution of COVID-19 vaccine across racial/ethnic groups	1.00	0.44	0.73	0.95

Estimated probabilities of selecting 1 vs. 0 for each dichotomously coded latent class indicator. The classes were labeled using the estimated probabilities of perceived COVID-19 threat (PCT), Perceived healthcare discrimination (PHD), and perceived healthcare system inequities which were categorized as low: < 33%, medium: 33–66%, high: >66%.

### Racial/ethnic differences in latent class membership

Compared to students in Class 4 (reference group), students in Class 1 had higher odds of self-identifying as Hispanic or Latino, Non-Hispanic Asian, Non-Hispanic Black or African American, and Non-Hispanic Multiracial compared to Non-Hispanic White [OR: 7.51 (95% CI: 2.83–19.90), OR: 4.29 (95% CI: 1.90–9.66), OR: 4.51 (95% CI: 1.74–11.68), OR: 3.85 (95% CI: 1.37–10.82), respectively] (Table 3). Students' race/ethnicity did not vary between the remaining classes.

**Table 3:** Racial/ethnic differences in latent class membership.

	**Class 1**	**Class 2**	**Class 3**
	**High probabilities of PCT** **Medium probability of PHD** **High probabilities of PHSI** ***n* = 118 (27.3%)**	**Low-medium probabilities of PCT** **Low probability of PHD** **Medium probabilities of PHSI** ***n* = 73 (16.9%)**	**Low-medium probabilities of PCT** **High probability of PHD** **Low-high probabilities of PHSI** ***n* = 22 (5.0%)**
**Race/ethnicity**		
Hispanic or Latino	7.51 (2.83–19.90)	1.69 (0.48–5.96)	1.18 (0.22–6.37)
Non-Hispanic Asian	4.29 (1.90–9.66)	2.05 (0.87–4.82)	0.48 (0.06–3.75)
Non-Hispanic Black or African American	4.51 (1.74–11.68)	0.52 (0.12–2.29)	2.20 (0.44–11.14)
Non-Hispanic Multiracial	3.85 (1.37–10.82)	1.21 (0.31–4.76)	1.05 (0.08–13.96)
Non-Hispanic White (reference group)	–	–	–

Odds ratios (95% CIs) of class membership compared to Class 4 reference class [low-high probabilities of perceived COVID-19 threat (PCT), low probability of perceived healthcare discrimination (PHD), high probabilities of healthcare system inequities (PHSI)]. Model adjusted for gender, age, and household income. The classes were labeled using the estimated probabilities of perceived COVID-19 threat, perceived healthcare discrimination, and perceived healthcare system inequities which were categorized as low: < 33%, medium: 33–66%, high: >66%.

### Associations between latent class health-related perceptions and self-rated mental and physical health

**Figure 2:**
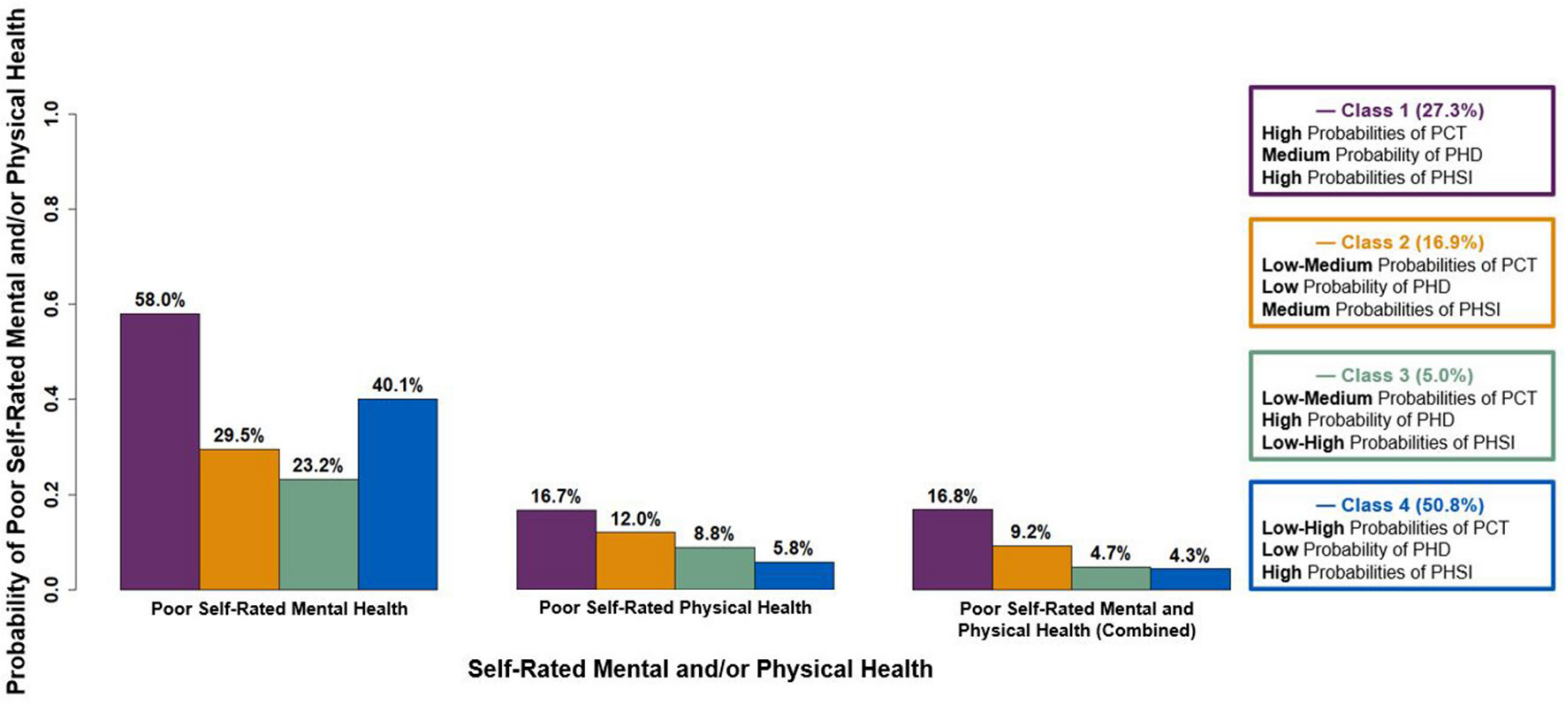
Probabilities of poor self-rated mental and/or physical health for each latent class.

The probabilities of poor self-rated mental and/or physical health for each latent class are presented in Figure 2. Compared to all other classes, students in Class 1 had higher odds of poor to fair mental health vs. good to excellent mental health [Class 1 vs. Class 2 OR: 3.31 (95% CI: 1.57–6.98), Class 1 vs. Class 3 OR: 4.77 (95% CI: 1.31–17.42), Class 1 vs. Class 4 OR: 2.08 [95% CI: 1.18–3.67)] (Table 4). Compared to Class 4, students in Class 1 had higher odds of poor to fair physical health vs. good to excellent physical health [OR: 3.74 (95% CI: 1.49–9.38)]. Class 1 had higher odds of poorer combined health compared to Class 4 (i.e., poor to fair mental and physical health vs. other categories of combined health that included good to excellent mental and/or physical health) [OR: 4.51 (95% CI: 1.78–11.44)]. In addition, Class 1 had higher odds of poorer combined health compared to all other classes (i.e., categories of combined health that included poor to fair mental and/or physical health vs. good to excellent mental and physical health) [Class 1 vs. Class 2 OR: 2.84 (95% CI: 1.37–5.90), Class 1 vs. Class 3 OR: 3.92 (95% CI: 1.14–13.43), Class 1 vs. Class 4 OR: 1.91 (95% CI: 1.08–3.37)] (Supplementary Table S3).

**Table 4:** Associations between latent classes and self-rated mental and physical health.

	**Class 1**	**Class 2**	**Class 3**
	**High probabilities of PCT**	**Low-medium probabilities of PCT**	**Low-medium probabilities of PCT**
	**Medium probability of PHD**	**Low probability of PHD**	**High probability of PHD**
	**High probabilities of PHSI**	**Medium probabilities of PHSI**	**Low-high probabilities of PHSI**
	***n* = 118 (27.3%)**	***n* = 73 (16.9%)**	***n* = 22 (5.0%)**
**Reference Class**			
**Class 2**			
Poor to fair mental health (vs. good to excellent mental health)	3.31 (1.57–6.98)	–	–
Poor to fair physical health (vs. good to excellent physical health)	1.74 (0.61–4.99)	–	–
Poor to fair mental & poor to fair physical health (vs. good to excellent mental health and good to excellent physical health, good to excellent mental health and poor to fair physical health, or good to excellent physical health and poor to fair mental health)	2.02 (0.72–5.73)	–	–
**Class 3**			
Poor to fair mental health (vs. good to excellent mental health)	4.77 (1.31–17.42)	1.44 (0.38–5.53)	–
Poor to fair physical health (vs. good to excellent physical health)	4.91 (0.47–51.24)	2.82 (0.25–31.63)	–
Poor to fair mental & poor to fair physical health (vs. good to excellent mental health and good to excellent physical health, good to excellent mental health and poor to fair physical health, or good to excellent physical health and poor to fair mental health)	4.74 (0.49–45.95)	2.34 (0.22–25.53)	–
**Class 4**			
Poor to fair mental health (vs. good to excellent mental health)	2.08 (1.18–3.67)	0.63 (0.31–1.27)	0.44 (0.13–1.50)
Poor to fair physical health (vs. good to excellent physical health)	3.74 (1.49–9.38)	2.15 (0.67–6.86)	0.76 (0.08–7.62)
Poor to fair mental & poor to fair physical health (vs. good to excellent mental health and good to excellent physical health, good to excellent mental health and poor to fair physical health, or good to excellent physical health and poor to fair mental health)	4.51 (1.78–11.44)	2.23 (0.63–7.82)	0.95 (0.09–9.74)

Odds ratios (95% CIs) comparing self-rated mental health, self-rated physical health, and self-rated mental and physical combined health across classes. Models adjusted for gender, age, and household income. The classes were labeled using the estimated probabilities of perceived COVID-19 threat (PCT), perceived healthcare discrimination (PHD), and perceived healthcare system inequities (PHSI) which were categorized as low: < 33%, medium: 33–66%, high: >66%.

## Discussion

The present study revealed racial/ethnic differences in latent class membership of health-related perceptions in a sample of college students. Students in Class 1 were more likely to identify as Hispanic or Latino, Non-Hispanic Asian, Non-Hispanic Black or African American, and Non-Hispanic Multiracial vs. Non-Hispanic White (compared to Class 4, reference group). Students in Class 1 had high probabilities of perceived COVID-19 threat, medium probability of perceived healthcare discrimination, and high probabilities of perceived U.S. healthcare system inequities and were more likely to report poorer mental and physical health compared to Class 4.

These findings are consistent with research that separately examined perceived COVID-19 threat, healthcare discrimination, and U.S. healthcare system inequities as predictors of mental and physical health outcomes (6, 14, 34–38). The present study examined the constructs collectively as indicators of underlying perceptions of COVID-19 threat, healthcare discrimination, and U.S. healthcare system inequities and found that distinct classes of these perceptions were associated with students' mental and physical health. In the COVID-19 context, much of the existing literature on college students' health-related perceptions examined the influence of perceived COVID-19 threat on preventive behaviors (39), therefore, this study extends prior research by focusing on the association between these health-related perceptions and self-rated mental and physical health. This extension has wide-reaching implications given that self-rated health is a rich, complex construct previously associated with biomarkers of stress, chronic disease, and mortality (40).

In addition, this study suggests potential processes underlying racial/ethnic differences in latent class membership and mental and physical health. Given that perceived COVID-19 threat was previously associated with greater stress and/or anxiety (41, 42), it is possible that higher perceived COVID-19 threat contributed to greater stress, and in turn, poorer mental and physical health for students in Class 1 compared to students in other classes with lower perceived COVID-19 threat. Moreover, since students in Class 1 were more likely to belong to minoritized racial/ethnic groups, their higher perceived COVID-19 threat may have been due to national racial/ethnic disparities in COVID-19 rates (43) as suggested in recent studies (15). The present study aligns with prior suggestions that racial/ethnic structural inequalities and national COVID-19 disparities may contribute to increased stress among college students (15), particularly for students from minoritized racial/ethnic groups. However, there is also recent evidence that students who identified as female and Black, Indigenous, and/or students of color had lower perceived stress over the course of the COVID-19 pandemic (44). Given the mixed evidence on racial/ethnic disparities in college students' health (5, 14, 24, 25), it is important for future studies to continue examining stress processes related to perceived COVID-19 threat and racial/ethnic differences in these relationships.

Furthermore, consistent with evidence in general adult populations (45, 46), students in Class 1 were more likely to perceive healthcare discrimination and report poorer mental and physical health compared to Class 4. Students in Class 1 may have expected a greater likelihood of interacting with healthcare providers due to their higher concerns over contracting the COVID-19 virus. Given that anticipated discrimination was previously associated with increased stress among African American and Latina American college students (47, 48), higher probability of healthcare discrimination for students in Class 1 may have resulted in greater stress and poorer mental and physical health compared to those in Class 4. Again, since students in Class 1 were more likely to belong to minoritized racial/ethnic groups, these findings may help explain recent evidence of racial/ethnic disparities in college students' mental health (5, 24).

Lastly, students in Classes 1 and 4 had relatively equal probabilities of perceiving U.S. healthcare system inequities. It is possible, however, that perceived racial/ethnic disparities in health inequity were more stressful for students in Class 1 as their greater likelihood of belonging to minoritized racial/ethnic groups may have increased their perceived risk of experiencing these disparities compared to Class 4. Additionally, since individuals can experience stress from injustices committed against others within their same social group (49), it is also possible that students experienced stress due to racial/ethnic healthcare system inequities affecting others from their racial/ethnic groups. These proposed explanations suggest potential harms of structural inequalities on college students' stress and health, even for those who have not personally experienced disparities in COVID-19 services.

Several limitations should be considered. The study was cross-sectional, which limits the ability to establish causality and address bidirectionality (i.e., poorer self-rated health could influence health-related perceptions). In addition, study interpretations are based on health-related perceptions serving as stressors among college students, in turn affecting their mental and physical health. However, since this study did not directly measure stress, it is possible that alternative processes may explain differences in students' health. Additional research is needed to directly measure stress and examine its mediating role of health-related perceptions and self-rated mental and physical health. Furthermore, the study was conducted at one university with a relatively small sample of students who self-selected into the study. Additionally, students who selected their race as “Other” or identified as transgender, gender non-conforming, or selected gender as “not listed” were excluded due to small sample sizes, resulting in a lack of representation across all gender identities and racial/ethnic groups. These factors limit generalizability as the findings may not be representative of all college students in the U.S. Lastly, the survey was collected over a relatively long time period, and it is possible that the relationships between health-related perceptions and self-rated mental and physical health changed over the course of the study. To address this limitation, survey completion date was included as a covariate in the analysis to control for the potential effects of time. However, future studies may wish to further examine these potential relationships.

Despite these limitations, the study had many strengths. This study included both self-rated mental and physical health, which were previously understudied in prior literature, and examined these measures separately and in combination. Moreover, this study addressed multiple socio-ecological factors related to health-related perceptions (i.e., intrapersonal COVID-19 threat perceptions, interpersonal factors related to healthcare discrimination, and perceived structural issues in the U.S. healthcare system). The racial/ethnic group characteristics of the study sample were also highly reflective of the university's undergraduate population (50).

These findings highlight ways that the multilevel nature of health-related perceptions may contribute to racial/ethnic disparities in college students' health, particularly in relation to the COVID-19 pandemic. In addition to academic and social stressors (51), the present study suggests that COVID-19-related stressors can influence college students' mental and physical health and further emphasizes the unique needs of college students during the COVID-19 pandemic (7). Continued research in this area is warranted given worsening mental health among college students (52) and rising racial/ethnic disparities in college students' health due to COVID-19 health concerns (14, 15). Public health research may also consider the unique circumstances of COVID-19 that influence health-related perceptions [e.g., stronger alignment of perceived COVID-19 threat and objective COVID-19 risk among minoritized racial/ethnic groups compared to other diseases (53)]. This may present an opportunity to apply evidence from the COVID-19 pandemic to other chronic illnesses that disproportionately burden minoritized racial/ethnic populations. Together, this study contributes to a limited body of evidence on college students' perceptions of the U.S. healthcare system and suggests important ways that structural inequalities and racial/ethnic disparities in COVID-19 risk, healthcare discrimination, and concerns over U.S. healthcare system inequity may affect college students' health.

## Data availability statement

The raw data supporting the conclusions of this article will be made available by the authors, without undue reservation.

## Ethics statement

The studies involving human participants were reviewed and approved by the University of Maryland, College Park Institutional Review Board. Written informed consent for participation was not required for this study in accordance with the national legislation and the institutional requirements.

## Author contributions

JRF: conceptualization, data curation, formal analysis, methodology, project administration, and writing—original draft. JSS: data curation, formal analysis, project administration, and writing—review and editing. YJC, JN, and NTJ: writing—review and editing. ATF: conceptualization, supervision, and writing—review and editing. All authors contributed to the article and approved the submitted version.
