# Supplementary material for: College students' underlying perceptions of COVID-19 threat, healthcare discrimination, and healthcare system inequities associated with self-rated health across racial/ethnic groups in the U.S
Source: Front Public Health. 2023 Jan 6;10:1028344. doi: 10.3389/fpubh.2022.1028344 (PMC9853174; doi:10.3389/fpubh.2022.1028344)
Supplement: Supplementary file 1 [file Data_Sheet_1.pdf]

College students' underlying perceptions of COVID-19 threat, healthcare discrimination, and healthcare system inequities associated with self-rated health across racial and ethnic groups in the U.S.

Supplemental Material

Table S1. Latent Class Indicators

Items	Response Categories	n (%)
**Perceived COVID-19 threat**		
"You believe COVID-19 is serious and life threatening" *(perceived COVID-19 severity)*	1. Not true<br>2. Somewhat true<br>3. Very true	1: 15 (03.5%)<br>2: 76 (17.6%)<br>3: 335 (77.5%)<br>*missing: 6 (1.4%)*
"You are concerned about contracting COVID-19" *(perceived COVID-19 susceptibility)*	1. Not true<br>2. Somewhat true<br>3. Very true	1: 61 (14.1%)<br>2: 198 (45.9%)<br>3: 172 (39.8%)<br>*missing: 1 (0.2%)*
**Perceived healthcare discrimination**		
"When getting healthcare, how often has each experience happened to you because of your race or skin color?<br>• Treated with less courtesy than other people<br>• Treated with less respect than other people<br>• Received poorer services than other people<br>• Had a doctor or nurse act as if he or she thought you were not smart<br>• Had a doctor or nurse act as if he or she was afraid of you<br>• Had a doctor or nurse act as if he or she was better than you<br>• Felt like a doctor or nurse was not listening to what you were saying"	0. Never<br>1. Once<br>2. 2 or 3 times<br>3. 4 times or more<br><br>*Note: Participants reported frequency for each experience. Scores were then averaged.*	0: 311 (72.0%)<br>>0: 119 (27.5%)<br>*missing: 2 (0.5%)*
**Perceived U.S. healthcare system inequities**		
"How often have racial and ethnic minority patients with COVID-19 been treated unfairly by the U.S. healthcare system because of their race or ethnicity?" *(perceived treatment of COVID-19 patients from minoritized racial/ethnic groups)*	1. Never<br>2. Somewhat often<br>3. Very often	1: 48 (11.1%)<br>2: 206 (47.7%)<br>3: 175 (40.5%)<br>*missing: 3 (0.7%)*
"How true is it that racial and ethnic minority groups have less access to COVID-19 testing compared to Whites?" *(perceived access to COVID-19 testing for minoritized racial/ethnic groups)*	1. Not true<br>2. Somewhat true<br>3. Very true	1: 77 (17.8%)<br>2: 193 (44.7%)<br>3: 161 (37.3%)<br>*missing: 1 (0.2%)*
"How confident are you that the COVID-19 vaccine will be distributed fairly across racial and ethnic groups?" *(perceived distribution of the COVID-19 vaccine across racial/ethnic groups)*	1. Not confident<br>2. Somewhat confident<br>3. Very confident	1: 58 (13.4%)<br>2: 218 (50.5%)<br>3: 156 (36.1%)

Dichotomization of perceived healthcare discrimination: Responses to the 7 items in the perceived healthcare discrimination scale were averaged ("Never" (0), "Once" (1), "2–3 times" (2), "4 times or more" (3)) and dichotomized (mean scores of 0: no experiences of healthcare discrimination coded as 0; mean scores > 0: one or more experiences of healthcare discrimination coded as 1).

Dichotomization of perceived COVID-19 threat and U.S. healthcare system inequities: Responses were first ordered from low to high perceived COVID-19 threat and perceived U.S. healthcare system inequities and the frequency distributions of the response categories were examined for each indicator. Four of the five indicators had sufficient frequencies for each response level (frequency percentages greater than 5% as suggested in the literature[1]). Given that only 15 of 432 participants (3.5%) responded that COVID-19 was "not severe", the categories of not severe and somewhat severe were coded as 0 and "very severe" was coded as 1. The same coding scheme was applied to perceived COVID-19 susceptibility to maintain consistency across the two perceived COVID-19 threat items. The perceived U.S. healthcare system inequities had sufficient frequencies across response levels and were dichotomized according to binary recoding schemes used in previous literature (e.g., not true, sometimes true, often true[2]).

[1] *Muthén B, Christoffersson A: Simultaneous factor analysis of dichotomous variables in several groups. Psychometrika. 1981, 46:407-419*

[2] *Kim M., Winkler, C., Talley, S.: Binary item CFA of Behavior Problem Index (BPI) using Mplus: A step-by-step tutorial. The Quantitative Methods for Psychology. 2021, 17*

Table S2: Model fit indices

	SA-BIC	Entropy	LMR LRT	LMR LRT p-value	PB LRT	PB LRT p-value
**1-Class Solution**	2821.09	-	-	-	-	-
**2-Class Solution**	2662.66	0.72	175.20	<0.01	179.26	<0.01
**3-Class Solution**	2644.00	0.74	38.61	<0.01	39.50	<0.01
**4-Class Solution**	2642.75	0.81	21.58	0.06	22.08	<0.01
**5-Class Solution**	2649.66	0.86	13.62	0.19	13.94	0.23

SA-BIC: sample-size adjusted Bayesian Information Criterion; LMR LRT: Lo-Mendell Rubin likelihood ratio test; PB LRT: parametric bootstrapped likelihood ratio test. Stronger model fit indicated by lower SA-BIC, higher entropy, and p-values <0.05 for the likelihood ratio tests.

Table S3. Associations between latent classes and combined self-rated mental and physical health

	**Class 1**<br>**High** Probabilities of PCT<br>**Medium** Probability of PHD<br>**High** Probabilities of PHSI<br>**n=118 (27.3%)**	**Class 2**<br>**Low-Medium** Probabilities of PCT<br>**Low** Probability of PHD<br>**Medium** Probabilities of PHSI<br>**n=73 (16.9%)**	**Class 3**<br>**Low-Medium** Probabilities of PCT<br>**High** Probability of PHD<br>**Low-High** Probabilities of PHSI<br>**n=22 (5.0%)**
**Reference class**			
**Class 2**			
Poor to fair mental & poor to fair physical health (versus good to excellent mental health & good to excellent physical health, good to excellent mental health & poor to fair physical health, or good to excellent physical health & poor to fair mental health)	2.02<br>(0.72 – 5.73)	-	-
Poor to fair mental & poor to fair physical health, poor to fair mental health & good to excellent physical health, or poor to fair physical health & good to excellent mental health (versus good to excellent mental health & good to excellent physical health)	2.84<br>(1.37 – 5.90)	-	-
**Class 3**			
Poor to fair mental & poor to fair physical health (versus good to excellent mental health & good to excellent physical health, good to excellent mental health & poor to fair physical health, or good to excellent physical health & poor to fair mental health)	4.74<br>(0.49 – 45.95)	2.34<br>(0.22 – 25.53)	-
Poor to fair mental & poor to fair physical health, poor to fair mental health & good to excellent physical health, or poor to fair physical health & good to excellent mental health (versus good to excellent mental health & good to excellent physical health)	3.92<br>(1.14 – 13.43)	1.38<br>(0.39 – 4.94)	-
**Class 4**			
Poor to fair mental & poor to fair physical health (versus good to excellent mental health & good to excellent physical health, good to excellent mental health & poor to fair physical health, or good to excellent physical health & poor to fair mental health)	4.51<br>(1.78 – 11.44)	2.23<br>(0.63 – 7.82)	0.95<br>(0.09 – 9.74)
Poor to fair mental & poor to fair physical health, poor to fair mental health & good to excellent physical health, or poor to fair physical health & good to excellent mental health (versus good to excellent mental health & good to excellent physical health)	1.91<br>(1.08 – 3.37)	0.67<br>(0.34 – 1.34)	0.49<br>(0.15 – 1.57)

Odds ratios [95% CIs] comparing combined self-rated mental and physical health across classes. Models adjusted for gender, age, and household income. The classes were labeled using the estimated probabilities of perceived COVID-19 threat (PCT), perceived healthcare discrimination (PHD), and perceived healthcare system inequities (PHSI) which were categorized as low: <33%, medium: 33-66%, high: >66%.
